# Failure and deformation characteristics of shale subject to true triaxial stress loading and unloading under water retention and seepage

**DOI:** 10.1098/rsos.220530

**Published:** 2022-07-27

**Authors:** Shujian Li, Chongyang Wang, Dongming zhang, Beichen Yu, Kangde Ren

**Affiliations:** ^1^ Yunnan Phosphate Chemical Group Co., Ltd., Kunming, Yunnan 650600, People's Republic of China; ^2^ School of Resources and Safety Engineering, Chongqing University, Chongqing 400030, People's Republic of China; ^3^ State Key Laboratory of Coal Mine Disaster Dynamics and Control, Chongqing University, Chongqing 400030, People's Republic of China; ^4^ National Engineering and Technology Research Center for Development and Utilization of Phosphate Resources, Kunming, Yunnan 650600, People's Republic of China

**Keywords:** true triaxial stress, shale, water retention, seepage

## Abstract

A multi-functional true triaxial fluid-structure coupling system was used to conduct water retention and seepage tests of shale under true triaxial loading and unloading stress paths. The stress–strain evolution of shale specimens under different experimental conditions was obtained, and the corresponding deformation and strength were analysed. The evolution and failure characteristics of cracks in shale were obtained by CT scanning images before and after the experiment. The results show that the volumetric strain of shale specimen increases first, then decreases and finally continues to increase with an increase in deviatoric stress under water retention, indicating that the volumetric change has experienced a compaction-expansion-compacting. The *σ*-*ε*_1_ curve of the sample increases first and then decreases, while the deformation in the *σ*_2_ direction shows the repeated compression and expansion. In the seepage test, the permeability–strain curve can be divided into two parts before and after fracture according to the *σ*-*ε*_1_ curve. Before fracture, the compression velocity of the specimen in the loading direction exceeds the expansion velocity in the unloading direction, resulting in a decrease in volume and a decrease in permeability. With an increase in deviatoric stress, fractures occur inside the particles and continue to spread from the tip until the fractures break through the shale specimen. The pore fissure area increases and the permeability of the sample increases rapidly. In terms of fracture evolution, for the water retention test, dense tensile and shear cracks appear on the failure plane perpendicular to the *σ*_1_ and *σ*_3_ directions, and complex shear fracture network appears on the failure plane perpendicular to the *σ*_2_ direction. For the seepage test, heavy shear failure occurs throughout the original fracture of the sample. With an increase in the penetration depth, the fracture shape on the failure surface perpendicular to the *σ*_2_ direction gradually changes from single to complex.

## 1. Introduction

**Figure 1.**
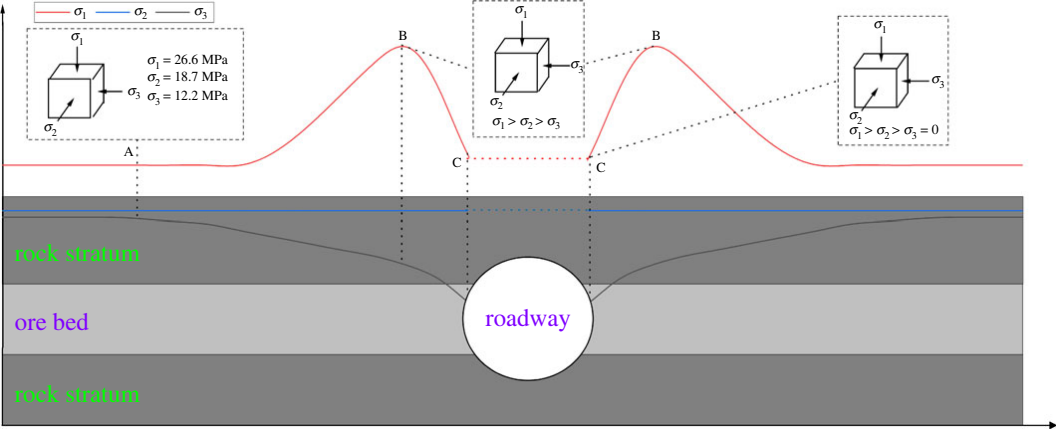
Stress environment of rock mass on both sides of roadway.

The rock mass of underground engineering is in the stress state of original rock before excavation. Mining stress is an important force that causes the deformation and failure of rock mass of underground engineering such as mining and tunneling. As shown in figure 1, during roadway excavation, the stress environment of surrounding rock mass will change significantly, which is manifested as unloading of horizontal stress and increasing of vertical stress. Therefore, the study of true triaxial stress loading and unloading on shale failure and deformation characteristics under water retention and seepage is of great significance to roadway support and surrounding rock control of underground engineering such as coal mines.

Many studies have been made of the mechanical properties of rock under true triaxial stress. Jiang *et al*. [1] established a constitutive model of rock elastoplastic damage based on the Mogi-Coulomb criterion and found that the model results were consistent with the test results. Zhang *et al*. [2] studied the rationality and reliability of true triaxial energy intensity criterion and concluded that the rock energy criterion is closely related to rock stress state and physical properties. He *et al*. [3] obtained the tight sandstone from the geological outgrowth of Shaximiao Formation in Sichuan Basin, and conducted hydraulic fracturing experiments of sandstone under true triaxial stress. Based on the apparent fracture characteristics and acoustic emission positioning results, they analysed the hydraulic fracture characteristics of the tight sandstone of Shaximiao Formation. Zhang *et al*. [4,5], using an indoor true triaxial geotechnical engineering simulation tester and fracturing pump pressure servo control system, the acoustic emission positioning system, with no confining pressure, confining pressure of sample analysis, found that influence the early stage of the hydraulic fracturing *in situ* stress difference coefficient of initial crack forming, horizontal ground stress difference coefficient is easier to form the initial crack, later period of crack propagation are greatly influenced by natural fracture. Gao *et al*. [6] considered the hydrostatic pressure of the vertical crack surface and the drag force of the parallel crack surface generated by high-pressure water, analysed the stress state of a single crack in compression shear and tension shear states, and deduced the stress intensity factor of the crack under the action of high-pressure water. According to the fracture extension mechanism of hydraulic fracturing, Qiu & Lu [7] modified the one-dimensional flow field of the original pseudo-three-dimensional model and established a new pseudo-three-dimensional flow field model based on the initial fracture geometry generated by the original pseudo-three-dimensional numerical model and the approximate two-dimensional flow field with radial flow emanating from the assumed virtual source. Wang *et al*. [8] developed a true triaxial shear seepage test system, which solved the problem that the previous shear seepage test was only affected by shear stress and normal stress, as well as the tightness of gas seepage in the shear process, and realized the experimental study of gas seepage under true triaxial shear deformation. The self-developed true triaxial gas-solid coupling coal seepage test system was used to carry out a coal adsorption seepage test with methane and helium gas as adsorption media and seepage media, and compared and analysed the mechanism of methane adsorption on coal deformation and permeability in a true triaxial stress environment [9]. In order to more truly simulate the stress state of coal rock under the heading face and other environments, Li *et al*. [10] studied the damage deformation and gas seepage of coal rock under the triaxial stress environment, and developed the true triaxial gas-solid coupling coal seepage test system. Bian [11] regards sandstone as the research object, using the multi-function fluid-structure coupling real triaxial test system, carried out true triaxial stress sandstone mechanical characteristics and law of seepage test, the different intermediate principal stress and minimum principal stress, different discharge rate and the gas pressure and unloading damage of sandstone and the influence of experimental research on seepage mechanics properties were investigated.

Nie *et al*. [12] established a true triaxial test system for coal seam gas, using the experimental system to study the true triaxial stress under the action of seepage of coal; the results show that the permeability of coal is a negative exponent along with an increase in volume stress is reduced when gas pressure gradient is constant. As the stress at constant volume, permeability of coal is increased with the increase of pressure gradient in parabolic rule changes. Zhang *et al*. [4,5] established a true triaxial compression numerical model, prefabricated cracks on numerical rock samples and studied the failure characteristics of fractured marble under true triaxial stress. Potyondy [13] used numerical simulation methods such as the discrete element method to conduct rock mechanics research and believed that the numerical simulation method was a powerful supplement to traditional research methods. Zhang *et al*. [4,5] used the parallel bonding model to first calibrate the mechanical properties of marble and then established a true triaxial compression numerical model, prefabricated cracks on numerical rock samples, and studied the failure characteristics of fractured marble under true triaxial stress. Zheng *et al*. [14] discussed the residual strength characteristics of Jinping marble under true triaxial compression and found that for higher minimum principal stress, the residual strength decreases first and then increases with the increase of intermediate principal stress, and for lower minimum principal stress, it gradually decreases with the increase of intermediate principal stress.

However, the research on true triaxial stress loading and unloading of shale under water retention and seepage is still insufficient. Therefore, this paper takes shale as the research object and adopts the multi-functional true triaxial fluid-solid coupling system to simulate the stress state under the mining depth of 200 m. Then, the loading and unloading tests of shale are carried out under the water retention and seepage conditions to analyse the mechanical and deformation characteristics of shale, and the crack evolution is obtained by CT scanning. It can provide some reference for roadway support and oil exploitation.

## 2. Sample characteristics and test methods

### 2.1. Specimen preparation

**Figure 2.**
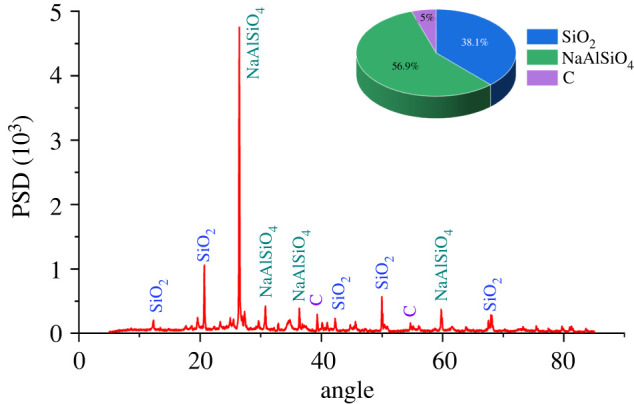
X-ray diffraction pattern of each group of samples.

The shale rock samples used in this study were taken from Yunnan Kunyang phosphate mine with a burial depth of 200 m. The selected shale was analysed by X-ray diffraction test for its internal composition, as shown in figure 2. As can be seen from figure 2, the main components of the shale sample used in this experiment are NaAlSiO_4_ $\left((1/2){\mathrm{Na}}_{2}O\cdot (1/2){\mathrm{Al}}_{2}0_{3}\cdot {\mathrm{SiO}}_{2}\right)$, SiO_2_ and a small amount of C. Among them, SiO_2_ content is the highest, at 60.5%. According to the proportion of each component, the molar ratio of each element is calculated as follows: *n*(O) : *n*(Si) : *n*(Na) : *n*(Al) : *n*(C) = 61 : 19 : 11 : 11 : 1. In addition to O, the non-metallic element with the highest proportion is Si, and the metal elements with the highest proportion are Na and Al.

The selected shale was cut and polished to make a cube specimen with a side length of 100 × 100 × 100 mm, and the flatness of the end face was controlled within 0.02 mm. At the same time, in order to avoid the impact of the end friction effect on the test, the mixed loading method (force-displacement loading) was adopted in this experiment to reduce the end friction effect. In addition, heat-shrinkable pipe is used to isolate the pressure head, pressurized oil and specimen, which reduces the end effect to a certain extent. Grease is also applied to reduce the friction between the heat-shrinkable tube and the specimen. The surface of the specimen is smooth without obvious joints and cracks. The shale specimen with perfect processing is shown in table 1. As can be seen from table 1, samples 1 and 2 have smooth surfaces and good texture. There are some natural fractures and joints on the surface of samples 3 and 4, which are related to the anisotropic structure of shale, but do not run through the rock mass and have little influence on the mechanical properties of shale.

**Table 1.** Well-processed shale sample.

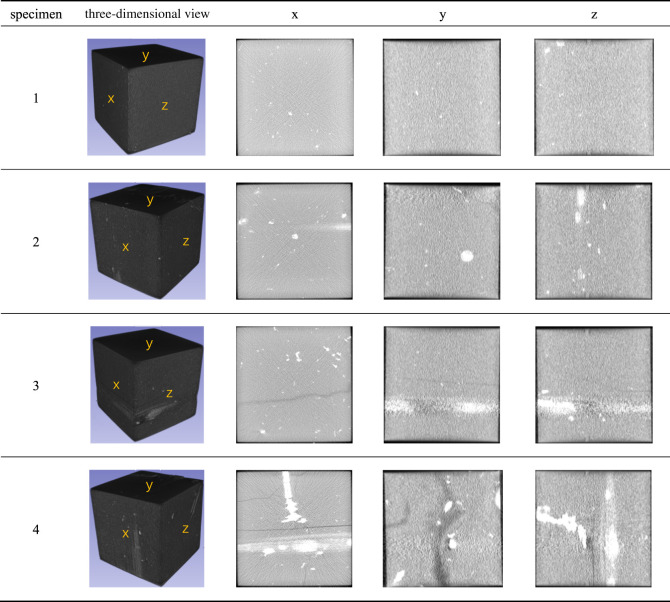

### 2.2. Testing equipment

**Figure 3.**
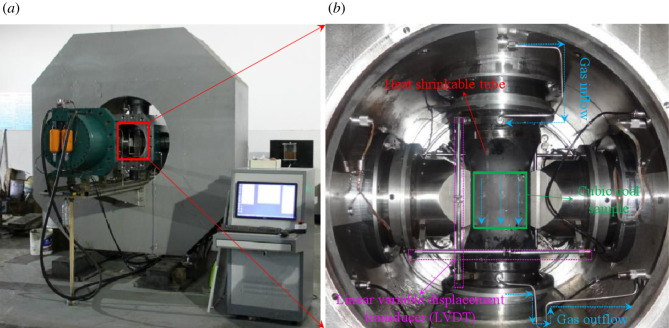
Multi-functional true triaxial fluid-solid coupling experimental system.

The water retention and seepage test of shale under true triaxial loading and unloading stress path adopts the ‘multi-functional true triaxial fluid-structure coupling experimental system’ independently developed by Chongqing University, as shown in figure 3.

The experimental device includes true three-axis pressure chamber, hydraulic oil pressure system, water or gas seepage system, true three-axis frame and disassembly platform guide rail, pressure, displacement and flow data monitoring system, AE signal monitoring system and so on. The pressure control method of the experimental device can ensure that the centre point of cubic coal rock remains unchanged during true triaxial loading. At the same time, the flexible and rigid loading adopted by pressure loading can effectively reduce the end effect. True triaxial compression range: the maximum confining pressure is 60 MPa, and the maximum load capacity of the three-way head is 6 × 10^3^ kN, 6 × 10^3^ kN, 4 × 10^3^ kN, respectively. The test system can carry out coal and rock mechanics, seepage and hydraulic fracturing experiments under different true triaxial stress paths. Meanwhile, the high-precision displacement sensor LVDT is used to test sample deformation, and the mass flowmeter can monitor the gas flow in the experimental process in real time.

### 2.3. Experimental method

**Figure 4.**
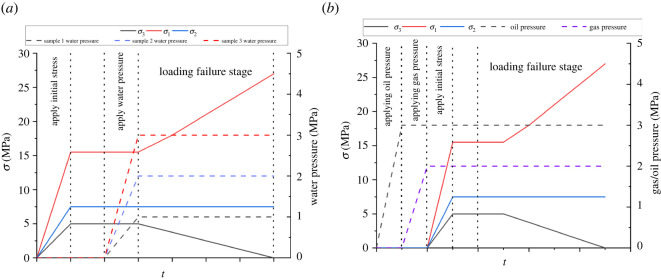
Loading path: (*a*) water conditions, (*b*) seepage.

To more accurately study the mechanical properties of shale under different water retention and seepage conditions, the initial *in situ* stress state of shale at 200 m depth is measured as follows: *σ*_1_ = 15.5 MPa, *σ*_2_ = 7.5 MPa, *σ*_3_ = 5.0 MPa. The sample method is shown in table 2, and the stress loading path is shown in figure 4.

**Table 2.** True triaxial loading stress path of shale.

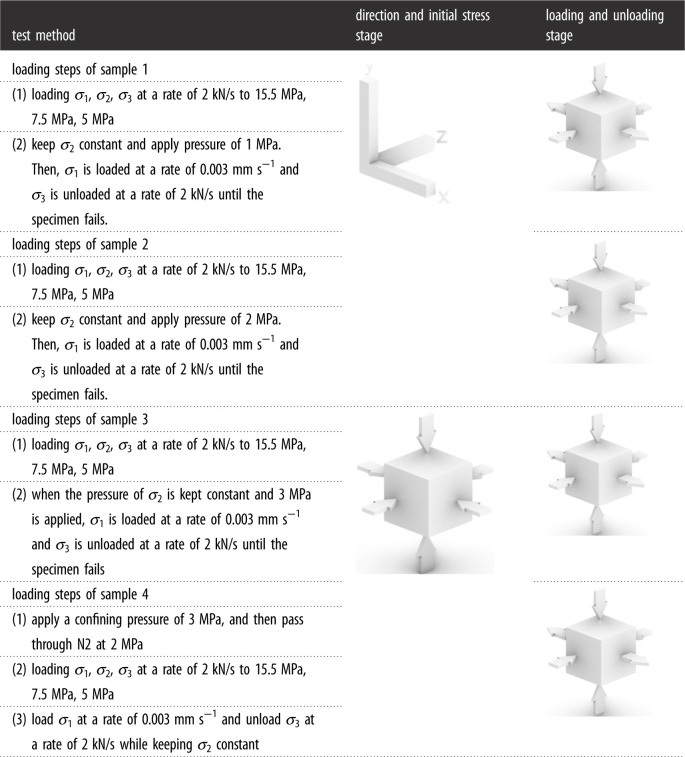

In the seepage test, Darcy's law is used to calculate shale permeability under true triaxial stress loading and unloading conditions [15], as shown in equation (2.1).

2.1 $$k=\frac{2q\mu LP_{2}}{A(P_{1}{\;}^{2}-P_{2}{\;}^{2})},$$

where *k* is permeability, m^2^; *q* is the gas seepage flow under standard condition, m^3^/s; *μ* is aerodynamic viscosity (19.9 µPa · s in this paper); *L* is the length of sample, m; *A* is the cross-sectional area of the sample, *m*^2^; *P*_2_ is atmospheric pressure, MPa; *P*_1_ is the air inlet pressure, MPa.

## 3. Test results and analysis

### 3.1. Deformation feature

**Figure 5.**
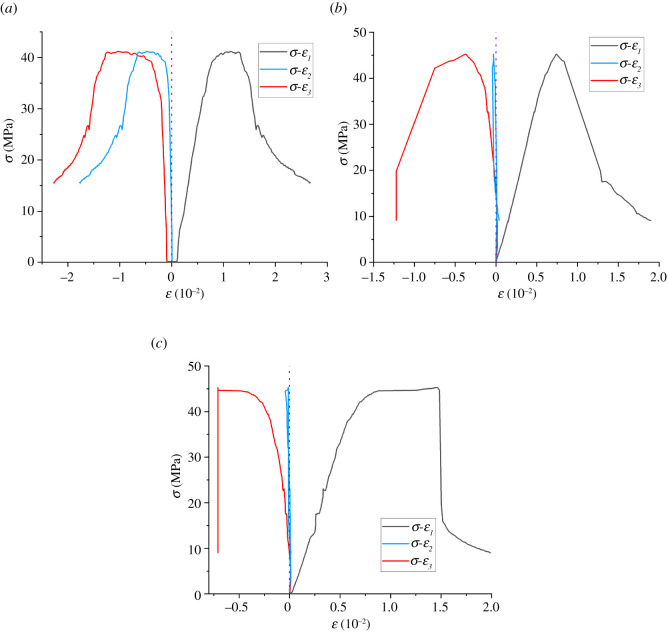
Deviatoric stress–strain curves of shale specimens under different water pressures. (*a*) Water pressure 1 MPa. (*b*) Water pressure 2 MPa. (*c*) Water pressure 3^MPa^.

There are certain differences in the stress distribution and deformation inside the surrounding rock of the roadway under different water pressures. The deformation characteristics of the shale specimen under different water pressure are deeply analysed, and the corresponding stress–strain curve is shown in figure 5.

As shown in figure 5, *σ* is the deviatoric stress, namely, $(1/3)\sqrt{{\left(\sigma_{1}-\sigma_{3}\right)}^{2}+{\left(\sigma_{1}-\sigma_{2}\right)}^{2}+\;{\left(\sigma_{2}-\sigma_{3}\right)}^{2}}$, *ε*_1_, *ε*_2_ and *ε*_3_ are the strains in the maximum, intermediate and minimum stress directions, respectively. It can be seen from figure 5 that the vertical strain value of the sample is the largest. When the water pressure is 1 MPa, 2 MPa and 3 MPa, the mean value of the vertical strain corresponding to the peak value of deviating stress is 0.76, 111.1% and 1333% larger than the mean value of the corresponding minimum horizontal strain and intermediate principal stress–strain. On the whole, the deviatoric stress-minimum horizontal strain curve is approximately axially symmetric with the deviatoric stress-vertical strain curve with *ε* = 0. Compared with *ε*_1_ and *ε*_3_, *ε*_2_ is significantly smaller, especially when the water pressure is 2 MPa and 3 MPa, the absolute value of *ε*_2_ is not more than 5‰, and the sample shows a trend of rebound at the stage after peak, resulting in the formation of a stress–strain closed loop. It can be seen from the above phenomenon that *σ*_2_ has little influence on the deformation characteristics of the sample, and the instability failure mechanism of the sample mainly depends on *σ*_1_ and *σ*_3_. The results confirm the reliability of the Mohr-Coulomb criterion in engineering practice.

In addition, according to the transverse comparison in figure 5*a–c*, the trend of deviating stress–strain curves of all samples is almost the same. The deviatoric stress-vertical stress curves of the three groups of samples all show four stages of compacts, linear elasticity, yield and post-peak. The maximum deviatoric stress of the samples under each water pressure is in the range of 40–50 MPa, indicating that the change of water pressure has little influence on the triaxial strength of the samples. The deviatoric stress-minimum horizontal stress curve of each sample shows another development trend. The curve is convex on the whole, and the whole curve is located in the tangent line of each part, showing the characteristics of convex shape. Different from the *σ*-*ε*_1_ curve, the *σ*-*ε*_3_ curve increases mainly after the deviator-vertical stress reaches its peak, which is due to the large deformation in this direction caused by the specimen failure. Then, with the continuous loading of the maximum principal stress direction, the *σ*-*ε*_3_ stress curve continues to increase, and the deformation of the *σ*-*ε*_2_ direction is not obvious. Investigate its reason is the sample after damage gradually lose bearing capacity, deformation is concentrated to *σ*_3_ direction. As the difference between *σ*_1_ and *σ*_2_ decreases, the deformation changes from expansion to compression in the direction of *σ*_2_. And with relatively large *σ*_1_ and *σ*_2_, it further increases the expansion in the direction of *σ*_3_.

**Figure 6.**
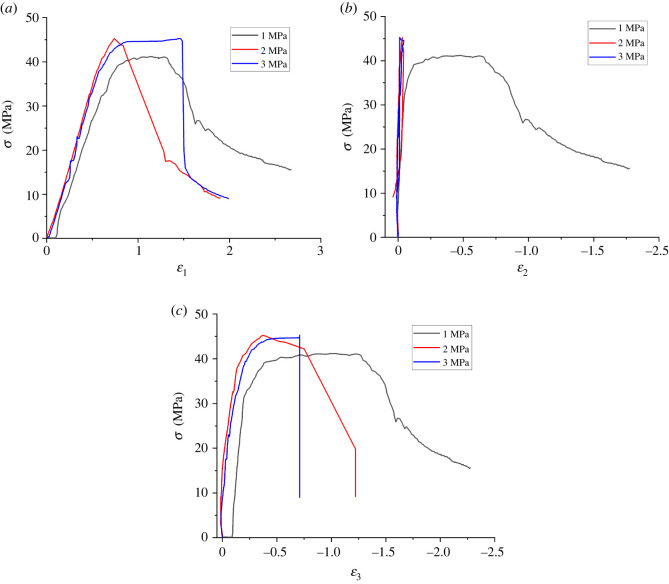
Comparison of deviatoric stress–strain. (*a*) *ε*_1_, (*b*) *ε*_2_ and (*c*) *ε*_3_.

Figure 6 shows the deviatoric stress–strain curves of shale in different principal stress directions. By figure 6, the *σ*_1_ and *σ*_2_ direction of partial stress–strain curve increases with the strain value in the direction of absolute value and presents a trend of decrease the first increases, but because of keep *σ*_2_, the strain in the direction of the absolute value is always less than *σ*_1_ direction, make the sample as a whole is always in a state of inflation. Except for the water pressure of 1 MPa (possibly due to the discreteness of the samples), the curves of the *σ*_3_ direction of the samples all show a stress–strain closed loop similar to the hysteresis loop, which is the result of repeated expansion and compression of the samples in *σ*_2_ direction.

In addition, according to the transverse comparison of figure 6*a–c*, it can be seen that under the same stress level, the maximum strain values of the samples in all directions are *ε* (*P* = 1 MPa) > *ε* (*P* = 2 MPa) > *ε* (*P* = 3 MPa). This indicates that the ductility of shale decreases gradually under high water pressure.

To further analyse the deformation characteristics of shale samples in all directions under different water pressures, the volumetric strain *ε*_V_ changes of samples 2 and 3 during loading were calculated, and the formula is as follows:

3.1 $$\varepsilon_{V}=\varepsilon_{1}+\varepsilon_{2}+\varepsilon_{3}.$$

**Figure 7.**
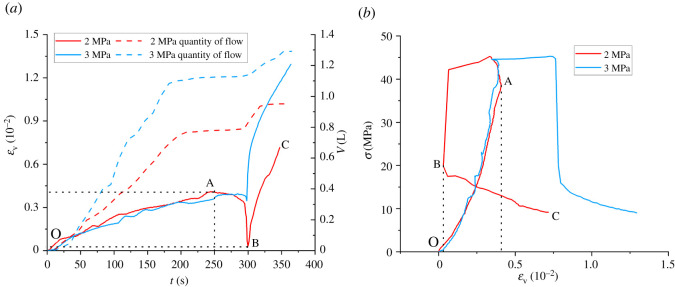
Relationship between deviatoric stress flow and volumetric strain. (*a*) *ε*_V_, *V*-t and (*b*) *σ-ε*_V_.

The volumetric strain of the sample is calculated by equation (3.1), and the volumetric strain–time curve and deviatoric stress–volumetric strain curve of the sample are drawn when the water pressure is 2 MPa and 3 MPa, as shown in figure 6. In the figure, *V* is the volume of flow through the water-retaining sample. It can be seen from figure 7 that the volumetric strains of both samples increase first, then decrease and then continue to increase with time. Since the curves of the two samples have similar trends when the water pressure is 2 MPa and 3 MPa, the curve of 2 MPa is taken as an example to analyse the variation characteristics of the volumetric strains:

(i) in the OA segment, namely *t*∈[0, 250], the volume strain of the sample increases gradually with the extension of time, but the increase rate decreases gradually, especially after *t*∈[230, 250], the curve gradually flattens out. It also corresponds to the OA segment in figure 7*b*. In figure 7*b*, at this stage, the *σ*-*ε*_V_ curve has two stages of compaction and linear elasticity, which is similar to the conventional stress–strain curve. The increase of volumetric strain corresponds to the decrease of sample volume, indicating that the sample is in the compaction stage.
(ii) in AB, namely *t*∈[250, 300], the volume strain of specimen with the extension of time to start showing a decreasing trend, *σ*_1_ of specimen near the peak, due to *σ*_3_ direction continues to uninstall, sample is gradually reduce the peak intensity, and with the increase of *σ*_1_, the internal fissure development unceasingly, the original sample new fractures appear gradually, which promote the overall expansion deformation of the sample, resulting in the increase of the sample volume in the direction of *ε*_3_.
(iii) when *t*∈[300, 350] reaches the peak strength, the sample gradually loses its bearing capacity. Currently, the displacement in the direction of *σ*_1_ continues to increase, resulting in the rapid collapse of the sample in this direction. At the same time, due to the constant discharge of *σ*_3_, the horizontal stress difference increases quickly, with samples in the *σ*_2_ direction rapidly expanding and destructive (this phenomenon is also visible in figure 5), and in the *σ*_1_ and *σ*_2_ directions at a faster rate than the *σ*_3_ direction of expansion rate, causing the sample volume to again show a decreasing trend as a whole.

Meanwhile, according to the analysis of flow data in figure 7*a*, the flow rate of the samples in the early OA stage is roughly the same, and the *V-t* curve is roughly linear. With the gradual compaction of the samples, the slope of the *V-t* curve gradually decreases and gradually presents a ‘yield’ state. After point A, the deviatoric stress continues to increase, the sample breaks and the flow curve surges again. Subsequently, with the continuous increase in volumetric strain, the sample compacts again and the flow gradually remains stable.

### 3.2. Infiltration characteristics

**Figure 8.**
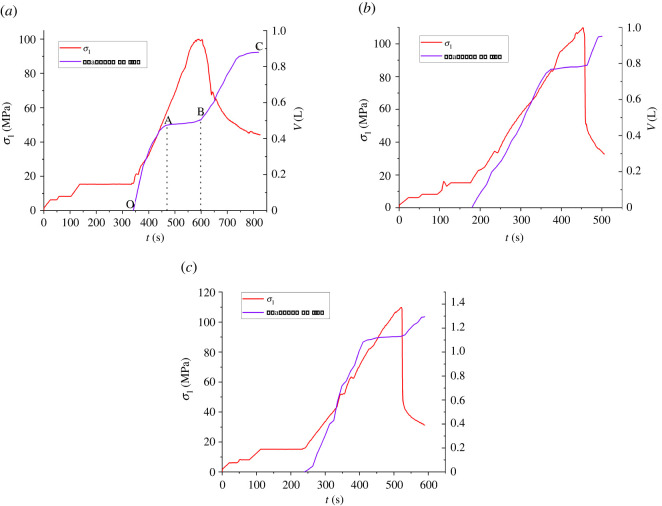
The relationship between water pressure and flow rate. (*a*) Water pressure 1 MPa, (*b*) water pressure 2 MPa and (*c*) water pressure 3 MPa.

In a water retention test, flow data is also an important factor to reflect the mechanical properties of samples. Figure 8 shows the curves of the flow data and *σ*_1_ changing with time during the water retention test.

It can be seen from figure 8 that the final flow through the specimen when the water pressure is 1 MPa is 0.8792 L, 7.6% and 32% less than when the water pressure is 2 MPa and 3 MPa, respectively, indicating that the flow through the specimen increases with the increase of the water pressure. As the flow trend of the three groups of samples is similar, the sample with 1 MPa water pressure is taken as an example to analyse the flow trend of the sample, which can be roughly divided into the following three stages:

In the OA section, *σ*_1_ of the sample is low, the shale is in an elastic state, and the internal fractures do not increase significantly, so the influence is small, and the flow through the sample increases roughly linearly.

In section AB, with the increase of three-way principal stress, the primary cracks of the sample are gradually compacted, and the flow rate through the sample decreases gradually. The increasing trend of flow rate almost stops before fracture.

In the BC section, as *σ*_1_ continues to increase, the sample begins to enter the yield stage. With the expansion of the original fractures and the generation of new fractures, the fracture area of shale increases and the flow rate through the sample increases. With the failure of the sample, the flow through the sample increases rapidly along with the occurrence of macroscopic fractures.

**Figure 9.**
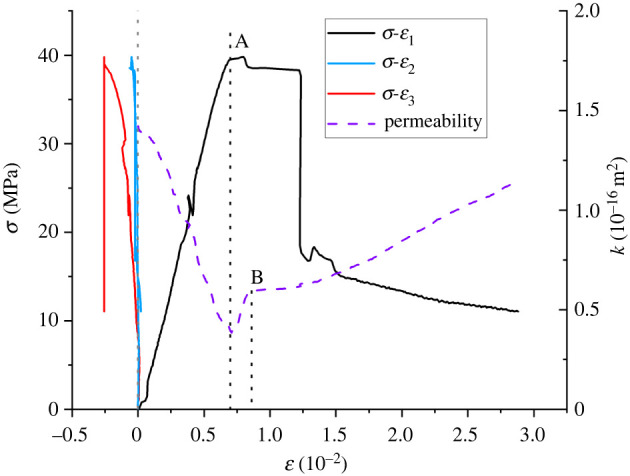
Stress and permeability–strain curve.

Figure 9 shows the stress permeability–strain curve of shale samples. It can be seen from figure 9 that the permeability of the sample increases first and then decreases with an increase in the vertical stress. Referring to the deviatoric stress–maximum horizontal strain curve, the permeability–vertical strain curve can be divided into two parts:

(i) Before the fracture of the sample, that is, before point A, the permeability decreases gradually, which is similar to the OB section in the fracturing test in figure 7. At this stage, the permeability decreases as a whole due to the compact three-direction compression and reduced volume of the sample.
(ii) After the sample reaches the peak strength, that is, after point A, fractures occur inside the particles due to the action of deviatoric stress and continue to expand from the tip until the fractures break through the shale specimen, and the fracture area increases rapidly. The permeability of samples increases rapidly, especially in section AB. When the sample is loaded to point B, shear slip occurs on the failure surface due to the expansion and connection of fractures, the volume strain of the sample further decreases, and the increasing rate of permeability is restrained.

**Figure 10.**
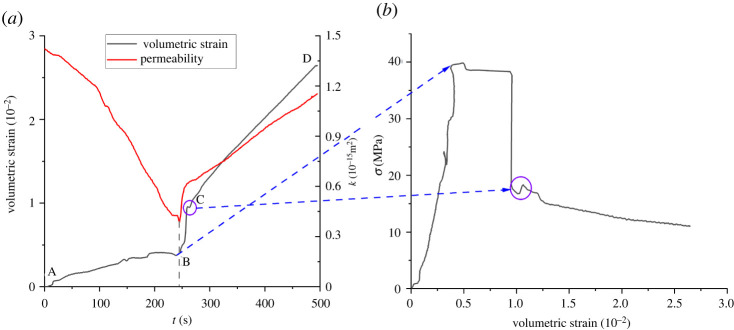
Deviatoric stress–volumetric strain–time curve of the sample.

Figure 10 shows the deviatoric stress–volumetric strain–time curve of the sample. Figure 10 shows that the volumetric strain of the sample is positively correlated with time on the whole. By analysing the variation characteristics of the volumetric strain, it can be seen that

(i) In the AB segment, that is, *t*∈[0, 245], the volumetric strain of the sample increases gradually with time, but the rate of increase decreases gradually. Especially in the t∈[200, 245], the curve growth stagnates and the sample volume even increases slightly. At this stage, the deviatoric stress is in a state of increase; the sample due to stress causes the primary fracture closure, and permeability continues to decrease.
(ii) In the BC segment, namely *t*∈[245, 270], the sample rapidly loses its bearing capacity due to fracture and deviatoric stress drops. Fractures in the sample keep expanding and penetrating, and even slip on the failure surface, resulting in the rapid increase of both permeability and volumetric strain.
(iii) In the CD segment, that is, *t*∈[270, 500], is the residual stress stage of the sample. With the continuous loading in the direction of maximum principal stress, the new fractures dislocation closed, and the growth rate of permeability and volumetric strain were restrained.

### 3.3. Strength characteristics

At present, the Mohr-Coulomb criterion and Drucker-Prager criterion are generally adopted for rock strength analysis. However, the Mohr-Coulomb criterion does not reflect the influence of intermediate principal stress on rock strength, so it is not applicable to the description of rock strength under true triaxial stress.

In order to improve accuracy, the Mogi-Coulomb criterion and Drucker-Prager criterion were used to compare and analyse the strength parameters of rocks under different water pressures in true triaxial stress.

The Mogi-coulomb criterion describes that there is a monotone increasing function relationship between octahedral shear stress and effective average normal stress [16,17], namely:

3.2 $$\tau_{oct}=a+b\sigma_{m,2},$$

3.3 $$\tau_{oct}=\frac{1}{3}\sqrt{{\left(\sigma_{1}-\sigma_{3}\right)}^{2}+{\left(\sigma_{1}-\sigma_{2}\right)}^{2}+\;{\left(\sigma_{2}-\sigma_{3}\right)}^{2}},$$

3.4 $$\mathrm{and}\;\;\sigma_{m,2}=\frac{\sigma_{1}+\sigma_{3}}{2},$$

$\tau_{oct}$ is octahedral shear stress; $\sigma_{m,2}$ is the average effective normal stress; *a* is the intercept of the fitting line of Mogi-Coulomb criterion; *b* is the slope of the line.

The relationship between Mogi-Coulomb parameters *a*, *b* and parameters of the Mohr-Coulomb criterion is as follows [18]:

3.5 $$a=\frac{2\sqrt{2}}{3}c\cos\varphi$$

and

3.6 $$b=\frac{2\sqrt{3}}{3}\sin\varphi .$$

The values of $\tau_{oct}$ and *σ*_*m*,2_ of shale samples are calculated by equations (3.3) and (3.4). The Mogi-Coulomb criterion equation of shale samples can be obtained by using the linear fitting method according to equation (3.6):

3.7 $$\tau_{oct}=11.1+0.67\sigma_{m,2}.$$

**Figure 11.**
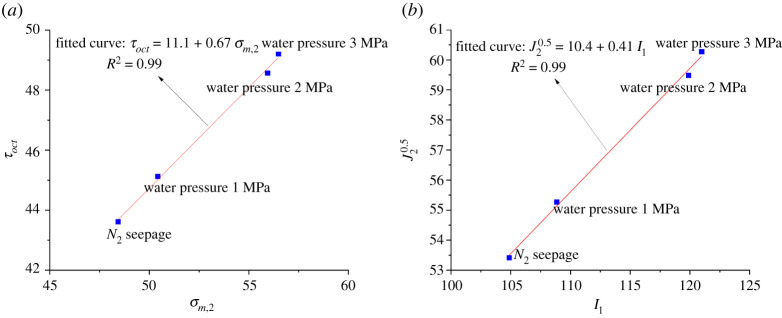
Mogi-Coulomb and Drucker-Prager guideline fitting curves. (*a*) Mogi-Coulomb criterion, (*b*) Drucker-Prager criterion.

The fitting curve is shown in figure 11*a*. The Mogi-Coulomb criterion *a* = 11.1, *b* = 0.67 is substituted into equations (3.6) and (3.7). The Mohr-Coulomb strength parameters of shale can be obtained as *c* = 14.5 MPa, *φ* = 35.5°.

The Drucker-Prager criterion is obtained based on the extension and extension of the C-M criterion and Mise criterion in plastic mechanics, and its criterion equation is

3.8 $$\sqrt{J_{2}}=\alpha I_{1}+k,$$

where *α* and *k* are test parameters; *I*_1_ and *J*_2_ are the first invariants of stress and the second invariants of stress skew, respectively.

Among them:

3.9 $$I_{1}=\sigma_{1}+\sigma_{2}+\sigma_{3},$$

and

3.10 $$J_{2}=\frac{1}{6}\left[{\left(\sigma_{1}-\sigma_{2}\right)}^{2}+{\left(\sigma_{2}-\sigma_{3}\right)}^{2}+{\left(\sigma_{3}-\sigma_{1}\right)}^{2}\right].$$

Considering the plane strain problem, the relationship between parameters of the Drucker-Prager criterion and Mohr-Coulomb criterion is as follows:

3.11 $$\alpha =\frac{2tan\varphi }{\sqrt{9+12tan^{2}\varphi }}$$

and

3.12 $$k=\frac{3c}{\sqrt{9+12tan^{2}\varphi }}.$$

The values of *I*_1_ and *J*_2_ of each sample are calculated according to equations (3.9) and (3.10). According to equation (3.8), the fitting equation of the Drucker-Prager criterion for the sample can be obtained by using the linear fitting method:

3.13 $$\sqrt{J_{2}}=0.41I_{1}+10.4$$

The fitting curve is shown in figure 11*b*, from which *α* = 0.41 and *k* = 10.4. According to equations (3.11) and (3.12), the Mohr-Coulomb strength parameters *c* = 14.8 MPa and *φ* =41.1° of the experimental shale can be obtained.

It can be seen from figure 11*a* and *b* that the fitting correlation coefficient *R*^2^ of the Mogi-Coulomb strength criterion and Drucker-Prager strength criterion reached 0.99, and the values of the Mohr-Coulomb strength parameters *c* and *φ* calculated by the two criteria were also close to each other. The results show that the two strength criteria are suitable for describing the failure strength characteristics of shale under water retention and seepage under true triaxial loading and unloading path.

### 3.4. Failure characteristics

The failure characteristics and fracture evolution law of rock are an important basis for analysing the failure mechanism of rock. CT scanning is carried out on the samples with water retention and seepage failure, and slices of their surface and interior are extracted for in-depth analysis of their failure characteristics. The results are shown in table 3. Because the failure characteristics of the three groups of hydraulic fracturing samples are similar, this paper mainly analyses the water retention conditions of 2 MPa and fracture evolution and failure characteristics of seepage samples. In table 2, *z* direction is *σ*_2_ direction, *x* direction is *σ*_3_ direction and *y* direction is the *σ*_1_ direction. Plane *y* of *x* direction is the section perpendicular to *y* direction obtained from the sample tangential to *x* direction.

**Table 3.** Failure characteristics.

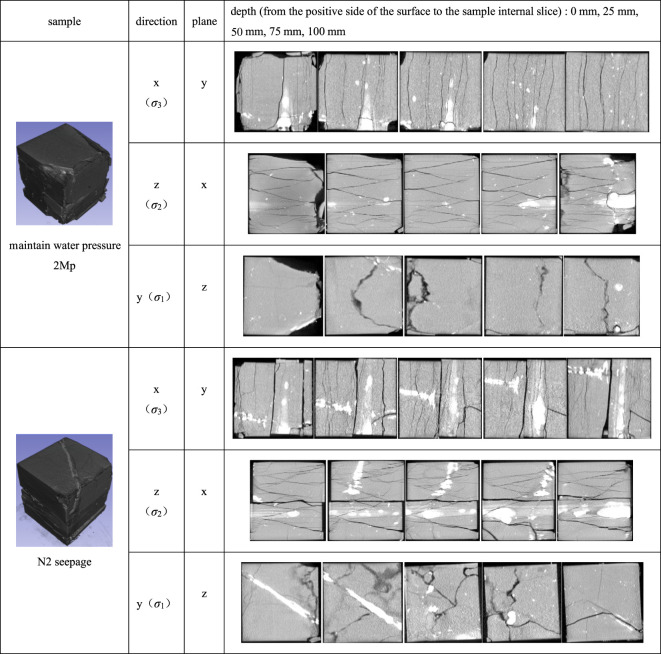

By table 3 longitudinal comparison, we can see the sample, epsilon *y* cutting length > epsilon *z* cut length > epsilon *x* cut long, among them, the *y* direction as *σ*_1_ direction, the direction of the length of the section is less than the other direction, shows that the direction of strain is mainly composed of compression, the *x* direction as *σ*_3_ direction, the direction of the length of the section, the largest strain show that the direction is mainly composed of inflation. These results are consistent with the above analysis.

By observing the failure characteristics of the water retention sample, it can be seen that dense vertical and inclined cracks appear on the failure plane perpendicular to the direction of maximum and minimum principal stress, representing the appearance of tensile fractures and shear fractures. Especially in the section perpendicular to the *σ*_1_ direction, the fracture density is the largest and the expansion is more obvious. Arc-shaped fractures appeared on the failure surface perpendicular to the *σ*_2_ direction, which oscillated repeatedly with an increase in the cutting depth, and a complex shear seam network appeared, corresponding to the folded cracks in the *σ*_2_ direction.

By observing the failure characteristics of the seepage sample, it can be seen that there are parallel fractures running through the samples on the failure planes perpendicular to the *σ*_1_ and *σ*_3_ directions. Combined with the tiny fractures already existing in sample 4 in table 1 before seepage, it can be seen that the heavy shear failure running through the original cracks of the sample occurs under the action of seepage. The fracture on the failure plane perpendicular to the direction of intermediate principal stress changes from single to complex fold and Y type with the increase of cutting depth.

### 3.5. Implications for field application

**Figure 12.**
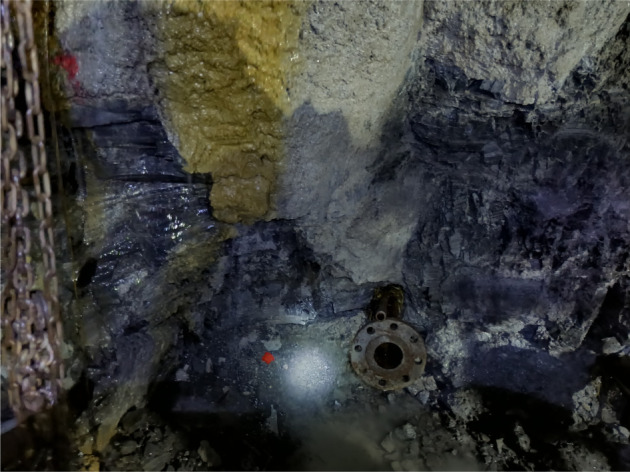
Roof permeable phenomenon.

Through a series of experiments, the deformation and failure characteristics of shale under water retention and seepage conditions are studied. The results show that different shear and tensile failures occur on the surface of samples under different water retention and seepage pressures. In the process of underground coal mining, permeability and ventilation of working face roof are unavoidable hydrogeological phenomena, as shown in figure 12. Once the flooding accident occurs, the normal production of the mining face will be affected, the working environment will be deteriorated, the drainage cost will be increased, and the service life of mechanical and electrical equipment and pipes will be shortened. In the worst case, it will cause surface flooding, mining area and well accidents, affect the development and utilization of coal resources, and cause the property loss of coal resources and even serious casualties. Therefore, it has important significance to the study of the mechanism of roof permeability and for exploring the deformation and failure characteristics of shale under different pressure and pressure conditions.

## 4. Conclusion

In this study, the multi-functional true triaxial fluid-solid coupling experimental system was used to conduct water retention and seepage tests on shale samples, and CT scanning of the samples before and after the test was conducted. The conclusions are as follows:

(i) The volumetric strain of samples increases first, then decreases and finally continues to increase under water retention, which exhibits compaction, expansion and re-compaction. The deviatoric stress–strain curve of the *ε*_1_ and *ε*_3_ directions increases first and then decreases with the increase of strain, while the *σ*-*ε*_2_ curves mostly show a stress–strain closed loop, due to the repeated compression and expansion of the sample in the intermediate principal stress direction. *σ*_max_ under each water pressure is in the range of 40–50 MPa, indicating that the change of water pressure has little influence on the triaxial strength of the samples.
(ii) The permeability of the sample increases first and then decreases with an increase in the vertical strain under seepage. Reference to deviatoric stress–strain curve and permeability–strain curve can be divided into before and bursts after two parts, before the break, sample by three-direction compression density, permeability decline on the whole, after the broken sample, due to its internal particle dispersion, crystal fracture, increase porosity increased, sample permeability increases quickly and gradually.
(iii) The Mogi-Coulomb and Drucker-Prager strength criteria were used to fit the shale samples under true triaxial loading. The values of *c* and *φ* of the strength parameters calculated by the two criteria were also close to the values of Mohr-Coulomb strength parameters, indicating that the two strength criteria are suitable for describing the failure strength characteristics of rock under true triaxial hydraulic fracturing and seepage.
(iv) Dense tensile cracks and shear cracks appeared on the failure plane perpendicular to the direction of *σ*_1_ and *σ*_3_ under water retention, and a complex shear fracture network appeared on the failure plane perpendicular to the direction of *σ*_2_. In the seepage test, heavy shear failure occurs through the original cracks of the sample on the failure plane perpendicular to the direction of *σ*_1_ and *σ*_3_, and the shape of the crack on the failure plane perpendicular to the direction of *σ*_2_ gradually changes from single type to complex folded and Y-shaped shear failure with the increase of the cutting depth. This indicates that the failure of the sample is mainly shear failure under water retention and seepage.

## Data Availability

The raw data accessibility of figures 5 to 11: Zhang, Dongming (2022), failure and deformation characteristics of shale under true triaxial stress loading and unloading under water retention and seepage, Dryad, Dataset, https://doi.org/10.5061/dryad.msbcc2g19 [19].
